# Genetic and clinical phenotypic analysis of familial stapes sclerosis caused by an *NOG* mutation

**DOI:** 10.1186/s12920-020-00843-5

**Published:** 2020-12-11

**Authors:** Rong Yu, Hongqun Jiang, Huihuang Liao, Wugen Luo

**Affiliations:** Department of ENT, First Affiliated Hospital of Nanchang University, Nanchang, 330006 Jiangxi People’s Republic of China

**Keywords:** Gene, Deafness, Stapes sclerosis

## Abstract

**Background:**

The noggin protein encoded by the NOG gene can interfere with the binding of bone morphogenetic protein to its receptor, thus affecting bone and joint development. The symptoms include abnormal skeletal development and conductive deafness.

**Methods:**

In a retrospective study, clinical data of the proband and her family members, including 8 people and 50 healthy normal controls, were collected. Second-generation sequencing was performed on peripheral blood samples from them.

**Results:**

The sequencing analysis indicated that in the proband, the *NOG* gene had a c.532T > C, p.C178R (cytosine deletion, NM_005450.6:c.532T > C), leading to an amino acid change. The proband's father, grandmother, second sister, and third sister also had this mutation, whereas family members with normal phenotypes did not have the mutation.

**Conclusion:**

Analysis of this family showed that the novel presentation of the c.532T > C, p.C178R mutation in the *NOG* gene resulted in syndrome-type autosomal dominant inheritance reflected in a mild clinical phenotype, which is of great importance for further studies of the clinical phenotype and pathogenesis of stapes sclerosis.

## Background

Conductive deafness is a type of hearing loss caused by lesions in the outer and middle ear. The passage of external sound waves into the inner ear is obstructed by pathological factors affecting the sound transmission system in the ear. Congenital causes are common malformation of the outer ear, hypoplasia of the tympanum, malformation of the ossicular chain, familial otosclerosis, etc. Acquired causes are commonly seen in external auditory canal obstruction by cerumen, foreign bodies, inflammation, scars, tumours, etc., or by tympanic membrane trauma, perforation, thickening and adhesion, middle ear effusion or pus, cholesteatoma, fracture of the auditory chain, ear sclerosis, tumour, etc. Stapes ankylosis is rare but can cause early conductive deafness [1–4]. It is difficult to differentiate from the common aetiology of conductive deafness and otosclerosis and is often overlooked, delaying treatment. Stapes ankylosis may be associated with skeletal dysplasia, such as osteogenesis imperfecta type I (OMIM 166200) [5–7]. Skeletal dysplasia is a common congenital malformation and a common phenotype in many inherited disorders.

Abnormal bone and joint development are the most common congenital malformations. Mutations in the *NOG* gene (OMIM 602991) on chromosome 17q21–q22 can lead to multiple autosomal dominant genetic diseases. The noggin protein encoded by the *NOG* gene can interfere with the binding of bone morphogenetic protein to its receptor, thus affecting bone and joint development, which may manifest as skeletal dysplasia and conductive deafness [8–11]. Skeletal dysplasia includes proximal interphalangeal joint fusion syndrome type A (OMIM 185800), multiple osseous junction syndrome type 1 OMIM 186500, tarsal and carpal joint fusion syndrome (OMIM 186570), short finger syndrome type B2 (OMIM 611377), and stapes stiffness with a wide thumb and toes (OMIM 184460) [8]. To improve the understanding of this rare disease, *NOG* gene analysis was performed in a family with stapes ankylosis in China.

Few studies have investigated the mechanism and correlation of stapes ankylosis in China, and a gold standard for pathological diagnosis is still lacking. Clinical diagn osis is mainly based on family history, genetic history, genetic testing, and phenotypic signs. Currently, surgery is the only specific and effective treatment. Therefore, a thorough understanding of the disease is important. In this study, we performed a clinical examination and genetic mutation analysis of a Han Chinese family with a suspected clinical manifestation of stapes ankylosis.

## Methods

Members of the family analysed in this study were from Jiangxi Province, China. The ten-member family, two of whom had died, included three generations. A detailed medical history was taken of the eight living family members, and all suspected patients underwent a detailed physical examination, including a specialist examination of the ear, height measurement, hand X-ray, and hearing tests. Other family members were identified as patients through detailed questioning. Peripheral venous blood was collected from the proband and other family members. The control group included venous blood samples from 50 normal controls in the molecular genetic database of our hospital. All adult subjects signed informed consent forms. The presence of other pathogenic variants for stapes ankylosis have been checked and ruled out.

The proband and her three sisters, father, mother, grandmother, and uncle were screened for testing. Peripheral venous blood samples were collected from the subjects with 3–5 mL of EDTA anticoagulant, and DNA was extracted using the Qiagen Genomic DNA Extraction Kit (Qiagen, Inc., Hilden, Germany) following the manufacturer’s instructions. The DNA was then quantified, tested for purity using a NanoDrop 1000 (Thermo Fisher Scientific, Waltham, MA, USA) and then stored at -80 °C. After purity testing, the extracted DNA was sent to the company's laboratory for relevant genetic testing. The target genes of the samples were captured by liquid-phase capture technology, and high-throughput sequencing was performed on the Illumina HiSeq 2000 platform (Illumina, San Diego, CA, USA) at an average depth of > 200X.

The criteria for selecting the variant as pathogenic is according to ACMG rules. The evaluation is as follows:Five members of this family were ill and mutated, achieving genotype-phenotypic co-isolation (PP1_M).SIFT; Polyphen2; The REVEL online software predicts the function of the mutant protein, suggesting that changes in the amino acid residue are harmful (PP3). Rare variation, Frequency-free information in gnomAD and other databases (PM2)Mutations of NOG gene will lead to corresponding phenotypes reported in this paper (PP4)0.3PM + 1PP = LP (suspected disease).

The sequencing results were compared to the Human Gene Mutation Database (HG19), Locus Specific Mutation Database (http://www.hgvs.org/dblist/glsdb.htm), dbSNP (v144), US National Center for Bioinformatics, SPIDEX Database, and other databases.

## Results

**Fig. 1:**
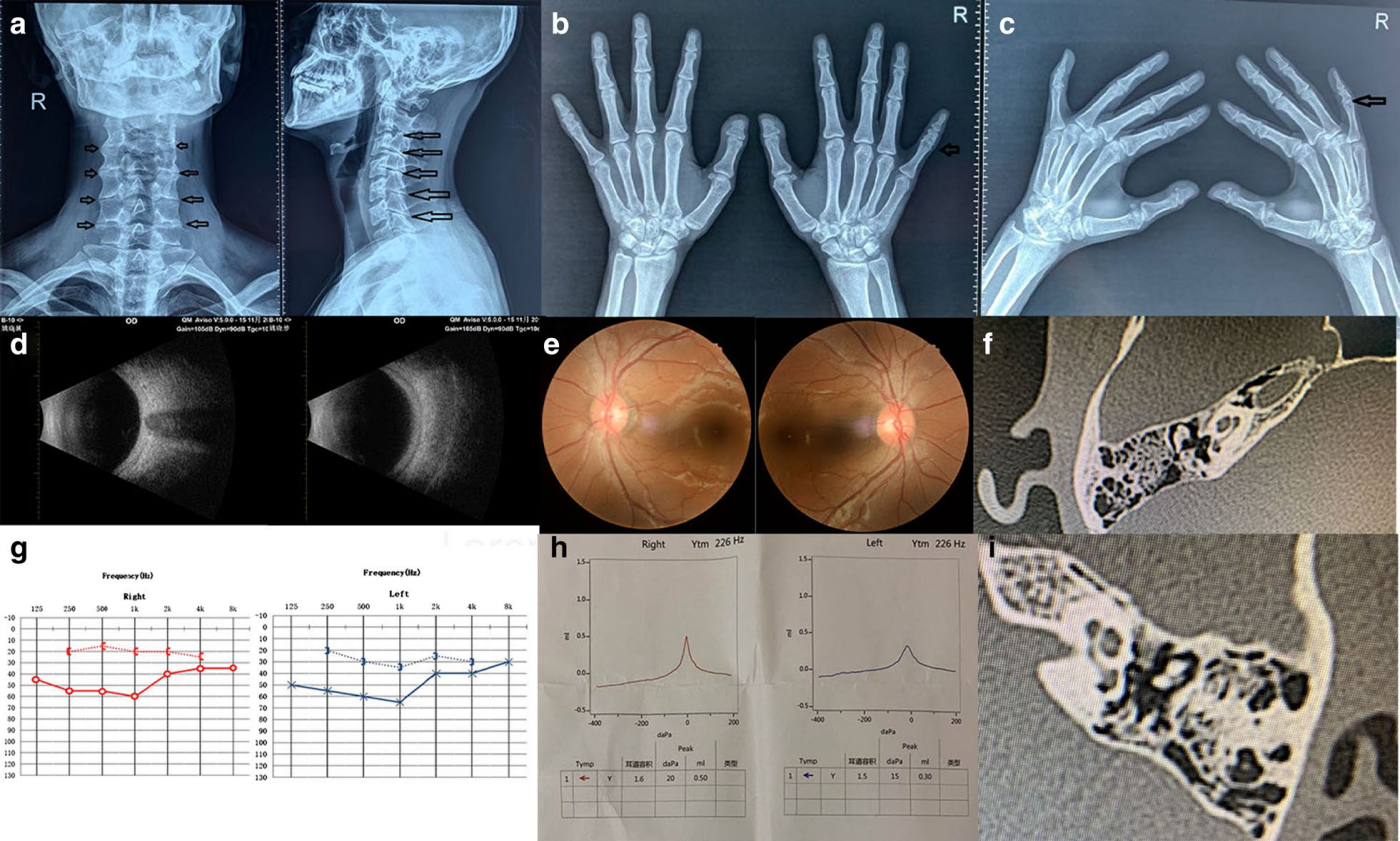
The audiological and physical examination of the probands **a** cervical verte bra degener ation by X-ray examination; **b**, **c** Joint space ambiguity by X-ray exam ination; **d** Thickened bulb in both eyes; The vitreous body of both eyes is punctate turbid by ultrasonography, **e** no obvious abnormality by fundus camera; **g** Binaural conductive hearing by PTA; **h** "A" type by acoustic immittance measurem ent; **f**, **i** The temporal bone CT

The proband’s parents denied inbreeding. The proband reported 4 years of progressive hearing loss with no history of otitis media, ototoxic drug use, or noise exposure. The proband was 168 cm in height and hyperopic, with broad thumbs and toes, difficulty bending little finger, and limited range of motion in the neck. Eardru ms on both sides were intact. The Rinne and 512 Hz tests using a tuning fork were negative, the Weber test was biased to the side with severe deafness, the Schwabach test showed bone conduction extension, and the Gelle test was negative, suggesting stapes plate fixation. Pure-tone audiometry indicated bilateral conductive deafness. No obvious abnormalities were found in acoustic conductivity tests or on ear computed tomography (Fig. 1). The clinical diagnosis was stapes ankylosis.

**Fig. 2:**
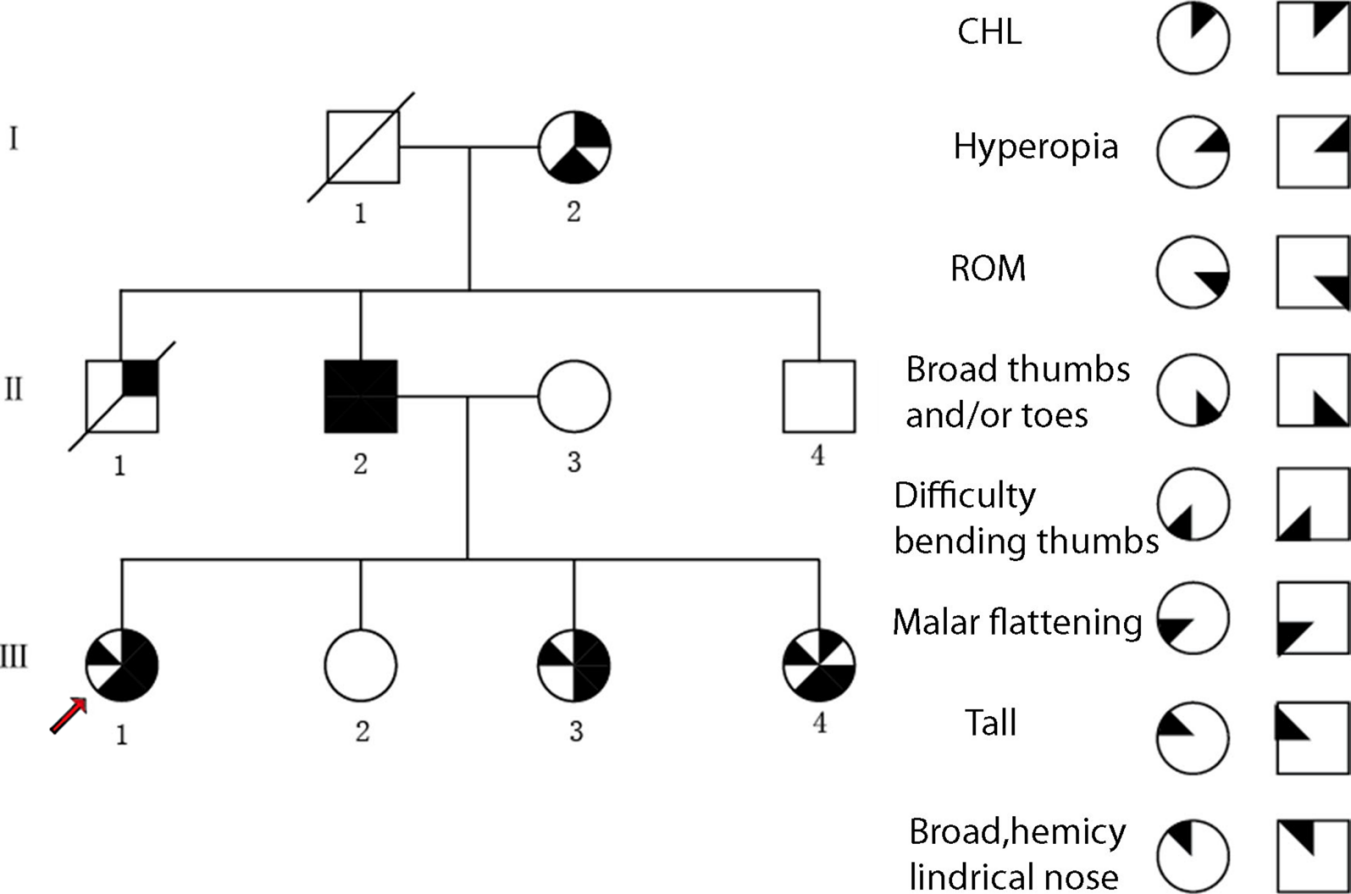
Pedigree of family. The arrow denotes the proband. Square symbols denote mal e patients, and circles denote female patients; solid black symbols denote affected indi viduals, and unfilled symbols denote unaffected individuals; each quadrant defines a phenotypic element or a set of phenotypic elements, and solid black quadrants indica te the presence of the corresponding phenotypic element(s)

Six family members had conductive hearing loss, stapes rigidity, hyperopia, and other phenotypic traits, such as broad thumbs and/or toes, difficulty bending little finger, decreased extremity and neck (range of motion) ROM. The proband had conductive hearing loss, hyperopia, broad thumbs and difficulty bending little finger, decreased extremity and neck ROM. The proband’s first sister had no obvious problems. The proband's second sister had the same symptoms as the proband except difficulty bending little finger. The proband’s third sister had the same symptoms as the proband except hyperopia. The proband's father showed the same symptoms as the proband, and her mother was congenitally deaf and mute. One of the proband's uncles, who had died in an accident, had hyperopia and conductive deafness. Another uncle was normal. The proband's grandmother had the same symptoms as the proband except decreased extremity and neck ROM. The patients in the family were tall except the proband's grandmother, and their walking function was normal. There was no other family history. Autosomal dominant genetic characteristics noted in this family are shown in Fig. 2.

**Fig. 3:**
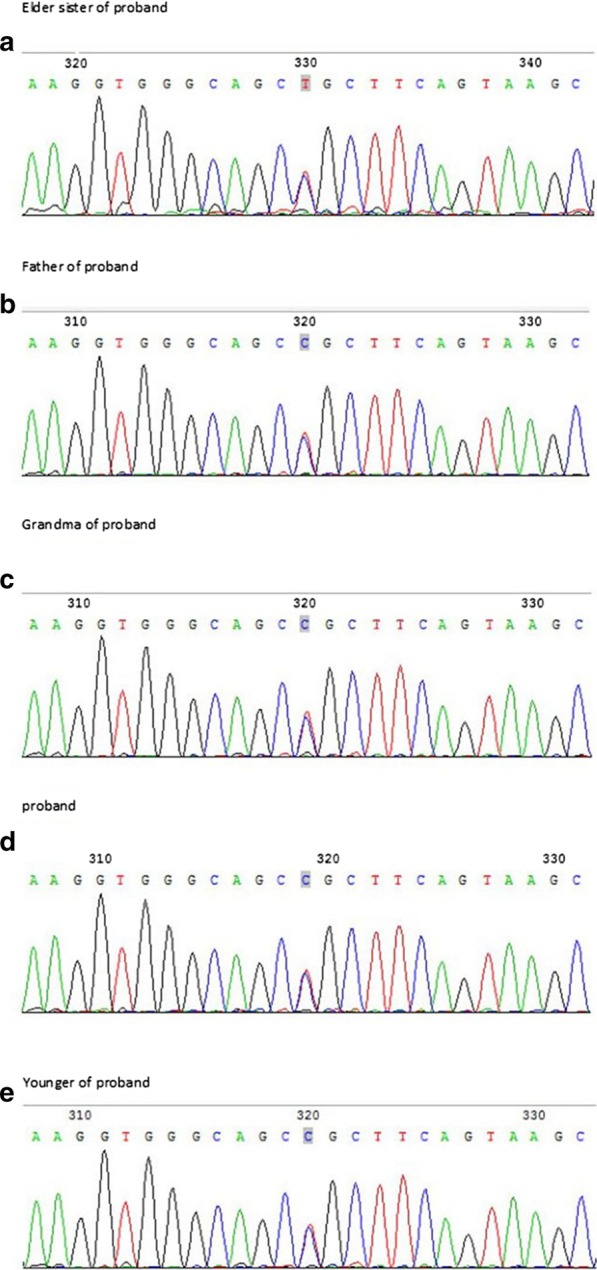
NOG C178R (chr17:54672116). **a** Sequencing results of NOG gene showed heterozygosity for the C178R mutation (shadow) of the elder sister of proband. **b** Sequencing results of NOG gene showed heterozygosity for the C178R mutation (shadow) of the father of the proband. **c** Sequencing results of NOG gene showed heterozygosity for the C178R mutation (shadow) of the grandmother of the proband. **d** Sequencing results of NOG gene showed heterozygosity for the C178R mutation (shadow) of the proband. **e** Sequencing results of NOG gene showed heterozygosity for the C178R mutation (shadow) of the younger sister of the proband

The sequencing results were compared to the Human Gene Mutation Database (HG19), Locus Specific Mutation Database (http://www.hgvs.org/dblist/glsdb.htm), dbSNP (v144), US National Center for Bioinformatics, SPIDEX Database, and other databases. The DNA sequencing analysis indicated that the *NOG* heterozygous mutation, namely, c.532T > C, p.C178R (code no. 532 nucleotide variation for cytosine by thymine, NM_005450.6:c.532T > C), was present in the proband’s father and grandmother. The proband’s two sisters also carried the mutation. To verify that this was the pathogenic mutation that caused stapes sclerosis, we screened 50 individuals with normal hearing from the Chinese population for *NOG* gene mutations. None of them had the mutation. Therefore, the c.532T > C, p.C178R mutation does not belong to the polymorphic site and is considered pathogenic (Fig. 3).

We searched the Chinese literature and PubMed database to find relevant literature on *NOG* gene mutations in a Chinese population. The results showed that nearly 40 mutations in the *NOG* gene have been identified, most of which are missense mutations located in the evolutionarily conserved region. Within this region is a functionally critical domain, suggesting a correlation between the region and the NOG protein. The c.532T > C, p.C178R mutation identified in this family has not been previously reported. The only clinical symptoms that were similar to those reported in our pedigree were those of Brown et al. [2], who showed stapes ankylosis accompanied by broad thumb, hyperopia, et al. In addition, the clinical characteristics of the affected individuals in this lineage are highly heterogeneous.

The variant analysis: the mutation causes the amino acid residue of Noggin protei n at C-terminal 178 to change from cysteine to arginine. The amino acid residue is located in this highly conserved region of cysteine—Knot B. SIFT; Polyphen2; REVE L online software predicts the function of the mutant protein, suggesting that changes in the amino acid residues are harmful. The mutation was not included in the normal population database: 1000 genomes, gnomAD and ExAC databases. This mutation achieves genotype-phenotypic co-isolation in this family.

Nog mutation site and clinical phenotype analysis: we searched literature database and PubMED data Library to find out the relevant literature of nog gene mutation. Table 1 lists the nog mutation sites reported so far Clinical phenotype [6, 12–18]. The results showed that the mutation site of nog was varied, the clinical features also vary. The clinical characteristics of the affected individuals in this family are highly heterog eneous.

**Table 1:** The mutation and clinical phenotypes of NOG

References	Nucleotide alteration	Annuo acid change	Proximal interphala ngeal joint fusion of finger	Proximal interphalangeal joint fusion of finger of toe	Conductive deafness	Cervical spine fusion	Distal interphalangeal joint fusion of finger	Unusual fecial appearance
[12]	No report	No report	+	**–**	**–**	**–**	**–**	**–**
[13]	No report	No report	+	**–**	+	**–**	**–**	Strabismus
[14]	c.551G > A	p.C184Y	+	**–**	**–**	**–**	**–**	**–**
[14]	c386T > A	p.L129X	+	**–**	+	**–**	**–**	**–**
[15]	c.450G > C	p.W150C	+	+	+	**–**	+	**–**
[16]	c.137T > C	p.L46P	+	**–**	**–**	**–**	**–**	Bifid nasal tip
[17]	c.559C > T	p.P187S	+	+	+	**–**	+	**–**
[6]	C.449C > T	p.R167C	+	+	**–**	**–**	+	**–**
[18]	C.391C > T	p.Q131X	+	+	+	**–**	**–**	Full forehead, Hyperopia

## Discussion

Congenital stapes ankylosis syndrome is rare, and the only finding consistent with this family's pedigree is described in a study by David J. Brown in an Italian family [2]. However, stapes ankylosis can cause early-onset conductive hearing loss. Congenital stapes ankylosis syndrome is difficult to distinguish from otosclerosis, the most common cause of conductive deafness, and is therefore prone to delayed diagnosis. The skeletal abnormalities it causes are mild, making the syndrome harder to recognize. Mutations in the *NOG* gene can lead to stapes ankylosis, conductive hearing loss, and joint damage. In this study, we report a familial stapes stiffness syndrome in one family with conductive hearing loss. Further genetic testing in this study revealed that the patient and the affected family members all carried the same *NOG* gene mutation. The clinical diagnosis was stapes ankylosis syndrome, and all patients in the family had conductive deafness. All of the patients were hyperopic except one. Some family members are unable to bend thumbs and broad thumbs and/or toes. Some of them have decreased extremity and neck ROM. The overall clinical phenotype of the patients in this family was relatively mild.

The C178R mutation in this family has not been reported before, but its clinical symptoms are similar to other NOG mutations [19–21]; however, previous studies did not report the ophthalmological findings in detail. The key features that differentiate SYM1 and SYNS1 from stapes ankylosis with broad thumbs and toes and hyperopia include a characteristic physiognomy, hyperopia, and the absence of cervical vertebral fusion and symphalangism.

The reported results for *NOG* mutation sites and clinical phenotypes show that the diversity of *NOG* is reflected in the diversity of mutation sites, the diversity of clinical manifestations, and the diversity of disease courses and prognoses. Ideally, genotype–phenotype relationship analysis is performed on a large sample of patients, but due to the diversity of gene mutations, patients with identical mutations are limited in number. Therefore, it is difficult to determine the relationship between the mutations and clinical phenotype. Bayat et al. proposed a new mutation in *NOG* in a Danish family in which cysteine 230 was replaced, but none of the affected family members suffered hearing loss [22]. Another study reported that mutations in cysteine 184 caused no indication of hearing impairment. In a reported family, both cysteine 184 and 230 were affected, resulting in stapes rigidity, hearing loss, and phalangeal dysfunction [23]. These findings confirm that mutations with the same NOG coding sequence can lead to different phenotypes and inter-family variation [6, 13–17, 21, 24, 25]. This suggests that disease expression can be independent of the location and type of *NOG* mutation. The spectrum of NOG mutations contains a variety of syndromes.

The reported effects of otological surgery for stapes fixation caused by NOG protein mutations vary [26–30]. In this study, surgical treatment was adopted in five ears (one patient), and hearing continued to improve after the operations. The follow-up period was 6 months. The patient's hearing improved by approximately 20 dB after surgery. Some reports describe progressive hearing loss due to bone regrowth 2–5 years after surgery. In this study, the number of patients was small, and it was difficult to determine statistical significance or make treatment recommendations. The outcome of stapedectomy may also depend on genetic variation. Our current results were all based on the pure-tone audiogram; the early audiogram did not assess the speech reception threshold. This threshold is also an important parameter and should be considered in the evaluation of surgical results.

## Conclusion

Conductive hearing loss is most often caused by otitis media or other middle ear lesions. *NOG* mutations can lead to early-onset conductive hearing loss. In combination with family history, genetic testing may help in identification. The initial use of clinical presentation and a family history of conductive deafness in the clinic require further evaluation. In the long term, the literature is inconsistent with regard to surgical outcomes, but the results of this study suggest that stapedectomy may be successful and can lead to lasting improvements in hearing. This family showed autosomal dominant inheritance related to the *NOG* gene, which is significant for understanding the pathogenic characteristics and clinical features of stapes ankylosis syndrome.

## Data Availability

All data supporting our results can be found in a published article. Current data on patients cannot be fully accessible in accordance with local research ethics protocols. However, if you are interested in this article, it may be available from the corresponding author.

## Abbreviations

NOG: Noggin
EDTA: Ethylene Diamine Tetraacetic Acid
SYM1: Symphalangism 1
SYNS1: Synostosis syndrome 1
ROM: Range of motion
